# Fusogenic-Oligoarginine Peptide-Mediated Delivery of siRNAs Targeting the CIP2A Oncogene into Oral Cancer Cells

**DOI:** 10.1371/journal.pone.0073348

**Published:** 2013-09-03

**Authors:** Liliana Cantini, Christopher C. Attaway, Betsy Butler, Lourdes M. Andino, Melissa L. Sokolosky, Andrew Jakymiw

**Affiliations:** Depatment of Craniofacial Biology and Center for Oral Health Research, Hollings Cancer Center, Medical University of South Carolina, Charleston, South Carolina, United States of America; University of Hong Kong, Hong Kong

## Abstract

Despite a better understanding of the pathogenesis of oral cancer, its treatment outcome remains poor. Thus, there is a need for new therapeutic strategies to improve the prognosis of this disease. RNA interference (RNAi) appears to be a promising therapeutic tool for the treatment of many diseases, including oral cancer. However, an obstacle for RNAi-mediated therapies has been delivery, in particular, the retention of small interfering RNAs (siRNAs) in endosomes and their subsequent degradation in lysosomes, resulting in inefficient gene silencing. Thus, the current study examined the feasibility of designing and utilizing a peptide, termed 599, consisting of a synthetic influenza virus-derived endosome-disruptive fusogenic peptide sequence and a stretch of cationic cell-penetrating nona(D-arginine) residues, to deliver siRNAs into oral cancer cells and induce silencing of the therapeutic target, CIP2A, an oncoprotein overexpressed in various human malignancies including oral cancer. Increasing the 599 peptide-to-siRNA molar ratio demonstrated a higher binding capacity for siRNA molecules and enhanced siRNA delivery into the cytoplasm of oral cancer cells. In fact, quantitative measurements of siRNA delivery into cells demonstrated that a 50∶1 peptide-to-siRNA molar ratio could deliver 18-fold higher amounts of siRNAs compared to cells treated with siRNA alone with no significant long-term cytotoxic effects. Most importantly, the 599 peptide-mediated siRNA delivery promoted significant CIP2A mRNA and protein silencing which resulted in decreased oral cancer cell invasiveness and anchorage-independent growth. Together, these data demonstrate that a chimeric peptide consisting of a fusogenic sequence, in combination with cell-penetrating residues, can be used to effectively deliver siRNAs into oral cancer cells and induce the silencing of its target gene, potentially offering a new therapeutic strategy in combating oral cancer.

## Introduction

It is estimated that about 40,000 new cases and approximately 8,000 deaths related to cancer of the oral cavity and pharynx will occur annually in the USA in 2012 [1]. Oral cavity cancer is currently ranked as the 6th most prevalent cancer globally, with squamous cell carcinomas of the oral mucosa being the most common type (∼90%) [2], [3]. Despite vast amounts of research and advances in the fields of oncology and surgery, the 5-year survival rate for oral cancer has only modestly improved in the last 30 years and its prognosis remains poorer compared to breast, colon, or prostate cancer [1]. Therefore, new therapeutic strategies are needed to improve the outcome of this disease.

RNA interference (RNAi) is a highly conserved post-transcriptional gene regulatory mechanism triggered by small, non-coding double-stranded RNA molecules that can specifically silence gene expression by either repressing translation and/or inducing mRNA degradation [4], [5]. Short double-stranded RNA molecules, known as small interfering RNA (siRNA) are functional molecules that in association with the RNA-induced silencing complex (RISC) mediate sequence-specific mRNA target selection and cleavage [6], [7], [8], [9]. The discovery that the introduction of chemically synthesized siRNAs into mammalian cells could efficiently induce sequence-specific inhibition of gene expression [6], made evident the therapeutic potential of harnessing RNAi as a means to specifically target and silence disease-causing genes. Subsequent preclinical experiments in animals and more recent clinical trials have further validated siRNAs as potent inhibitors of an assortment of disease-causing genes and as a promising new class of therapeutics [8], [10], [11].

Although the design of therapeutic-grade siRNAs has improved [8], [10], delivery still remains the single greatest obstacle towards the pervasive use of siRNAs for therapeutic applications [8]. Because therapeutic macromolecules are generally delivered through endocytosis [12], one of the major limiting steps for many delivery approaches, including siRNA delivery, is endosomal entrapment and subsequent degradation of the therapeutic cargo in lysosomes [11], [12], [13]. Thus, to enhance the intracellular bioavailability of siRNAs, effective strategies for endosomal escape are needed.

To transport the viral genome into the cytoplasm, animal viruses that are internalized via receptor-mediated endocytosis, utilize proteins with endosome-disruptive fusion peptide domain sequences to mediate destabilization of the host cell endosomal membrane [14]. The method by which these viral proteins destabilize endosomal membranes occurs in an acidification-dependent manner and has been mimicked by synthetic peptides, termed fusogenic peptides [15], [16]. In particular, several synthetic fusogenic peptides based on the N-terminal fusion domain of the HA2 subunit of the influenza virus hemagglutinin protein have proven to be effective at influencing gene transfer [15]. Among these fusogenic peptides, both the INF-7 peptide and its dimeric form diINF-7, have demonstrated their endosome disruptive capability by improving non-viral gene transfer systems and enhancing both the cytosolic delivery of immunoliposome-entrapped macromolecules and lipofectamine-mediated siRNA-induced gene silencing [15], [16], [17]. Moreover, co-incubation of chimeric peptides consisting of the HA2 endosome-disruptive peptide fused to cell-penetrating peptides (CPPs; cationic peptide vectors that can rapidly induce their own cellular internalization through different forms of endocytosis [18]), have also been demonstrated to enhance the endosomal escape of CPP-complexed cargo, consequently promoting stronger biological effects [12], [18], [19]. In one particular study, co-incubation of HA2-linked CPP peptides with CPP-complexed siRNAs was found to moderately improve the silencing of the targeted reporter gene [20].

Although the above described strategies have demonstrated improvements in siRNA-mediated silencing effects, several problems still remain that could limit the efficacy of fusogenic peptide-mediated cytosolic delivery of siRNAs. First, because the fusogenic or HA2-chimeric peptides were not complexed to the siRNAs, this approach did not guarantee that the peptides would co-localize within the same endocytic vesicles as the siRNA cargo [12]. Co-localization is required in order to release siRNA molecules from the endocytic vesicle [12]. Second, due to this lack of interaction between the peptides and siRNAs, it is possible that the peptides could escape the endosomes without seriously disrupting the endosomal membranes, ultimately leaving the siRNA cargo entrapped within the vesicle [12]. Thus, to circumvent these problems, this study aimed to design a chimeric peptide consisting of a fusogenic sequence linked to a CPP that would enable stable binding with siRNAs via electrostatic interactions and promote their intracellular delivery and endosomal escape, in order to induce the therapeutic silencing of a targeted oncogene in oral cancer cells. Because the INF-7 peptide is a more potent membrane-destabilizing peptide compared to the parent HA2 peptide and cationic nona(D-arginine) peptides are highly efficient CPPs capable of enhanced cellular uptake [12], [21], as well as delivery of siRNAs into tumors and brains of mice [22], [23], we designed a chimeric peptide, termed 599, that would harness both these properties within a single molecule to directly complex to and enhance the intracellular bioavailability of siRNAs. Here, we demonstrate that the 599 peptide effectively delivers siRNAs designed to target CIP2A, an oncoprotein overexpressed in human head and neck squamous cell carcinomas (HNSCCs), including oral squamous cell carcinomas (OSCCs) [24], [25], [26], into oral cancer cells, mediates efficient CIP2A silencing, and consequently inhibits oral cancer cell invasiveness and anchorage-independent growth.

## Results

### The 599 Peptide Effectively Binds and Delivers siRNAs into Oral Cancer Cells

**Figure 1:**
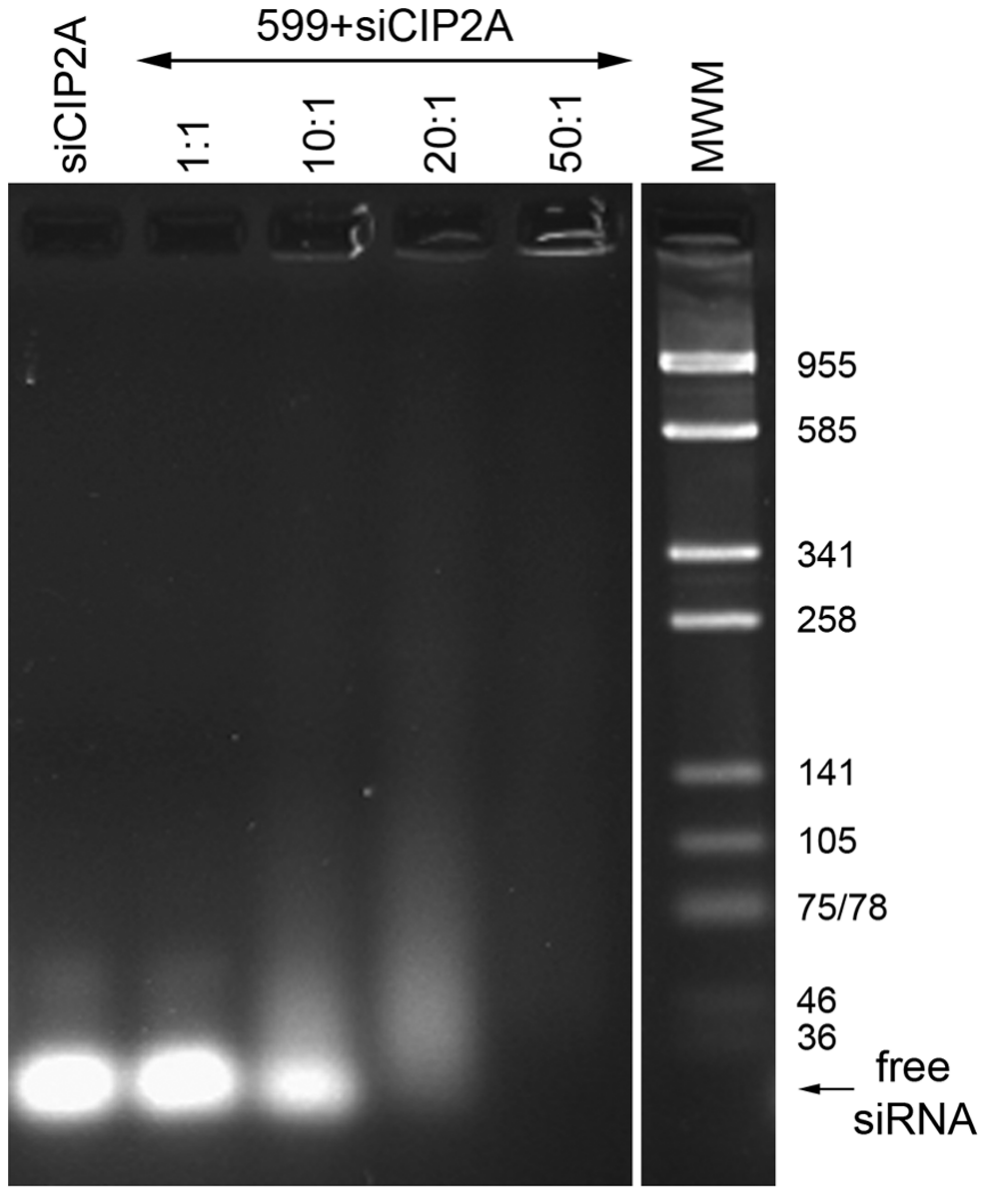
Optimization of 599 peptide binding to siRNAs. An ethidium bromide stained 4% agarose gel shift assay examining the ability of various amounts of the 599 peptide (ranging from 1 to 50-fold molar excess of siRNAs) to form complexes with siCIP2A. siCIP2A, siRNA targeting the CIP2A oncogene; MWM, molecular weight marker (the number of base pairs for each DNA fragment are shown).

In order to test whether the 599 peptide via its cationic nona(D-arginine) residues could effectively bind negatively charged siRNAs based on electrostatic interactions, an agarose gel shift assay was performed (**Fig. 1**). Using various amounts of the 599 peptide, ranging from 1 to 50-fold molar excess of siRNAs, it was demonstrated that the 599 peptide could indeed bind siRNA molecules designed to target CIP2A (siCIP2A) by retarding the siCIP2A starting at a peptide-to-siRNA molar (P:N) ratio of 20∶1, with no free siRNAs detectable in the gel at 50∶1. At P:N ratios of 10∶1, the peptide only had moderate effects on siRNA binding, and at 1∶1 it was completely ineffective.

**Figure 2:**
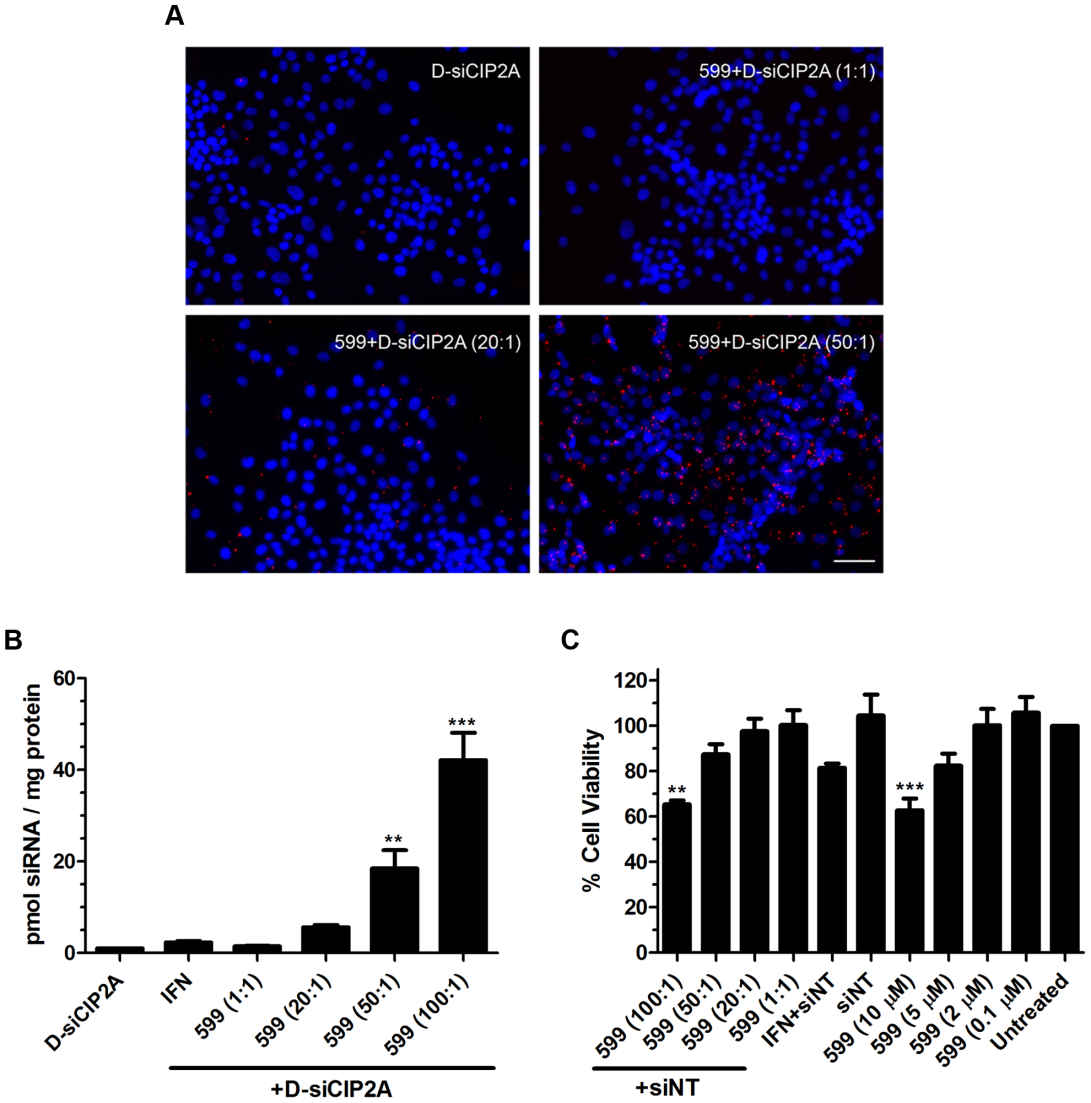
Optimization of 599 peptide-mediated delivery of siRNAs into oral cancer cells. (**A**) Fluorescence microscopy analysis of CAL 27 oral cancer cells incubated for 2 hours with DY547-conjugated siRNA targeting CIP2A (D-siCIP2A; red) alone or in complex with increasing amounts of 599 peptide (ranging from 1 to 50-fold molar excess of siRNAs). Nuclei (blue) were counterstained with DAPI. Scale bar: 50 µm. (**B**) CAL 27 cells incubated for 2.5 hours with D-siCIP2A alone or in complex with increasing amounts of 599 peptide (ranging from 1 to 100-fold molar excess of siRNAs). For comparison, the cells were also transfected using the commercial transfection reagent, INTERFERin™ (IFN). The amount of siRNA delivered into cells in pmol per mg of protein is reported with each treatment normalized to D-siCIP2A alone. Data are mean ± SEM of four separate experiments, where ***P<0.001, **P<0.01 compared to D-siCIP2A alone treated cells (ANOVA, Dunnett’s Multiple Comparison Test). (**C**) Assessment of long-term toxicity (as measured by a cell proliferation assay) of CAL 27 cells 48 hours post-treatment with either a non-targeting siRNA (siNT) alone, increasing concentrations of 599 peptide alone, or increasing amounts of 599 peptide (ranging from 1 to 100-fold molar excess of siRNAs) in complex with siNT. For comparison, the cells were also transfected with the commercial transfection reagent, IFN. Untreated cells were defined as 100% viable. Data are mean ± SEM performed in triplicate (n = 3), where ***P<0.001, **P<0.01 compared to untreated cells (ANOVA, Dunnett’s Multiple Comparison Test).

Upon demonstrating that the 599 peptide could bind to siRNAs at specific P:N ratios, we next examined the ability of the peptide to deliver fluorescently-labeled siRNAs into oral cancer cells by fluorescence microscopy analysis (**Fig. 2A**). Using a fluorescently-labeled siRNA, DY547-conjugated to siCIP2A (D-siCIP2A), in complex with increasing amounts of 599 peptide, ranging from 1 to 50-fold molar excess of siRNAs, it was observed that increasing the P:N ratio enhanced the delivery of siRNA molecules into CAL 27 oral cancer cells after two hours incubation. More specifically, the 50∶1 P:N ratio was demonstrated to have an increased ability at inducing siRNA uptake into cells compared to the 20∶1 P:N ratio which only had a moderate effect. Conversely, cells treated with D-siCIP2A alone or in complex with 599 peptide at a 1∶1 P:N ratio were incapable of effective siRNA uptake. Quantitative measurements of internalized D-siCIP2A into CAL 27 cells after 2.5 hours incubation also corroborated that increasing the 599 P:N ratios increased siRNA uptake into cells. In particular, the 50∶1 and 100∶1 P:N ratios delivered approximately 18 and 42-fold significantly higher amounts of D-siCIP2A, respectively, compared to cells treated with D-siCIP2A alone (**Fig. 2B**). As a comparison, the commercial transfection agent INTERFERin™ (IFN) only delivered approximately 2-fold higher amounts of D-siCIP2A, compared to cells treated with D-siCIP2A alone. Of note, although the 100∶1 P:N ratio was capable of delivering the highest amount of siRNAs into cells, it induced significant long-term cytotoxic effects as measured by using a non-targeting siRNA (siNT) control (**Fig. 2C**). In particular, the 100∶1 P:N ratio significantly reduced the proliferation of cells 48 hours post-treatment compared to untreated cells. Conversely, the 50∶1 P:N ratio had no significant effects on long-term cytotoxicity. As a result, based on these findings the 50∶1 P:N ratio was used for further characterization and experimentation.

### Characterization of the 599 Peptide-siRNA Complex

**Figure 3:**
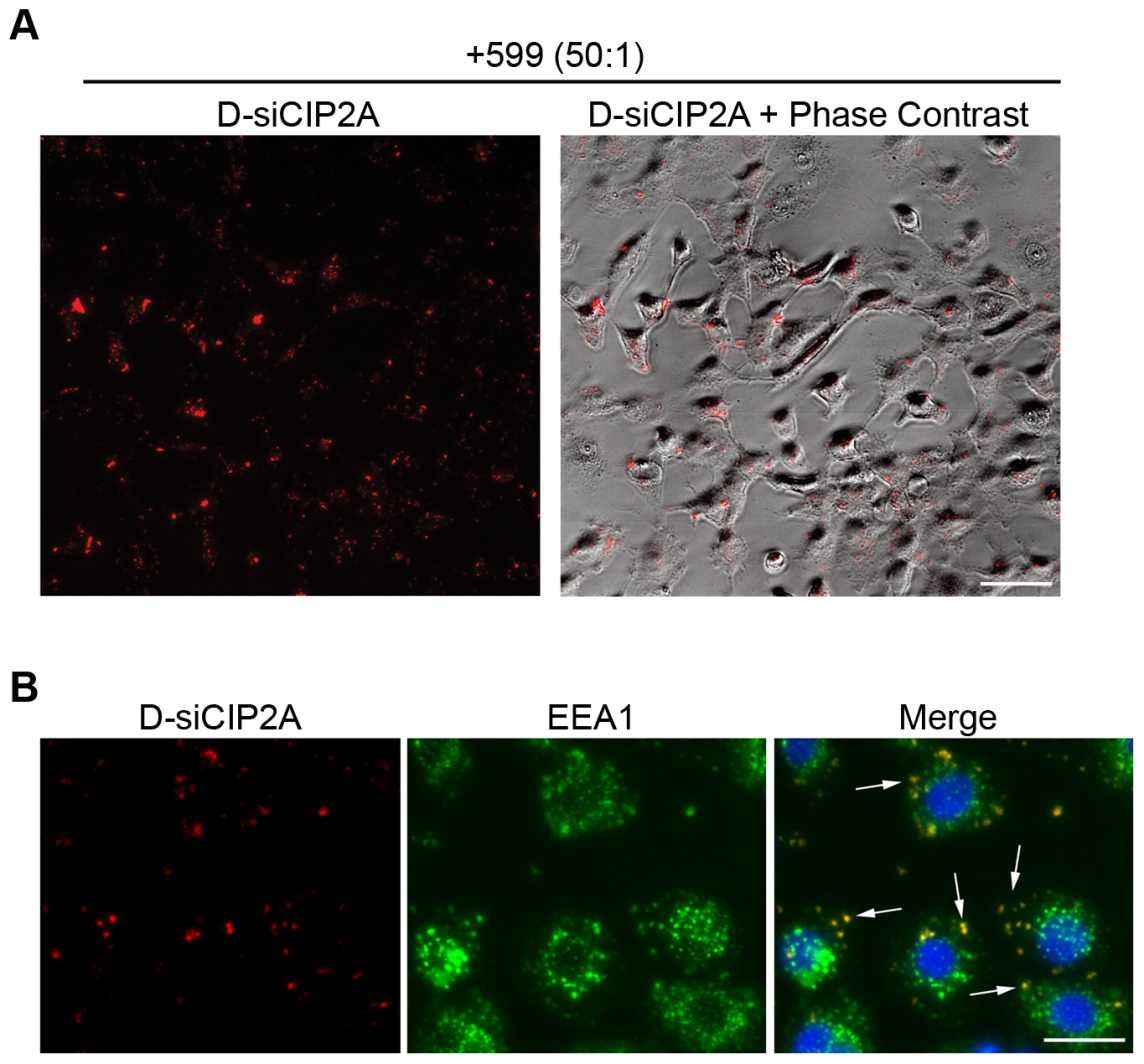
The 599 peptide mediates delivery of siRNAs into cells via endocytosis. (**A**) Fluorescence microscopy analysis and overlay of a phase contrast image of live CAL 27 oral cancer cells incubated for 2 hours with DY547-conjugated siRNA targeting CIP2A (D-siCIP2A; red) complexed with the 599 peptide at a 50∶1 peptide-to-siRNA molar ratio. Scale bar: 50 µm. (**B**) Indirect immunofluorescence microscopy analysis of CAL 27 oral cancer cells incubated for 2 hours with D-siCIP2A (red) complexed with the 599 peptide at a 50∶1 peptide-to-siRNA molar ratio. Endosomes (green) were stained using a rabbit monoclonal anti-EEA1 antibody. Nuclei (blue) were counterstained with DAPI. The co-localization of D-siCIP2A with endosomes (yellow) is observed in the merged panel and is emphasized by arrows. Scale bar: 25 µm.

To gain insight into the mechanism of cell entry of the 599/siRNA complex, the subcellular localization of the 599/D-siCIP2A complex was further examined in live cells by fluorescence microscopy coupled with phase contrast images (**Fig. 3A**). Upon closer inspection of the internalized D-siCIP2As, it appeared based on the extensive punctate uptake pattern of siRNAs that the 599/D-siCIP2A complex was being endocytosed. Co-staining of fixed cells with the early endosomal marker EEA1, confirmed the colocalization of D-siCIP2As with endosomal vesicles (**Fig. 3B**), thus indicating an endocytic internalization mode.

**Figure 4:**
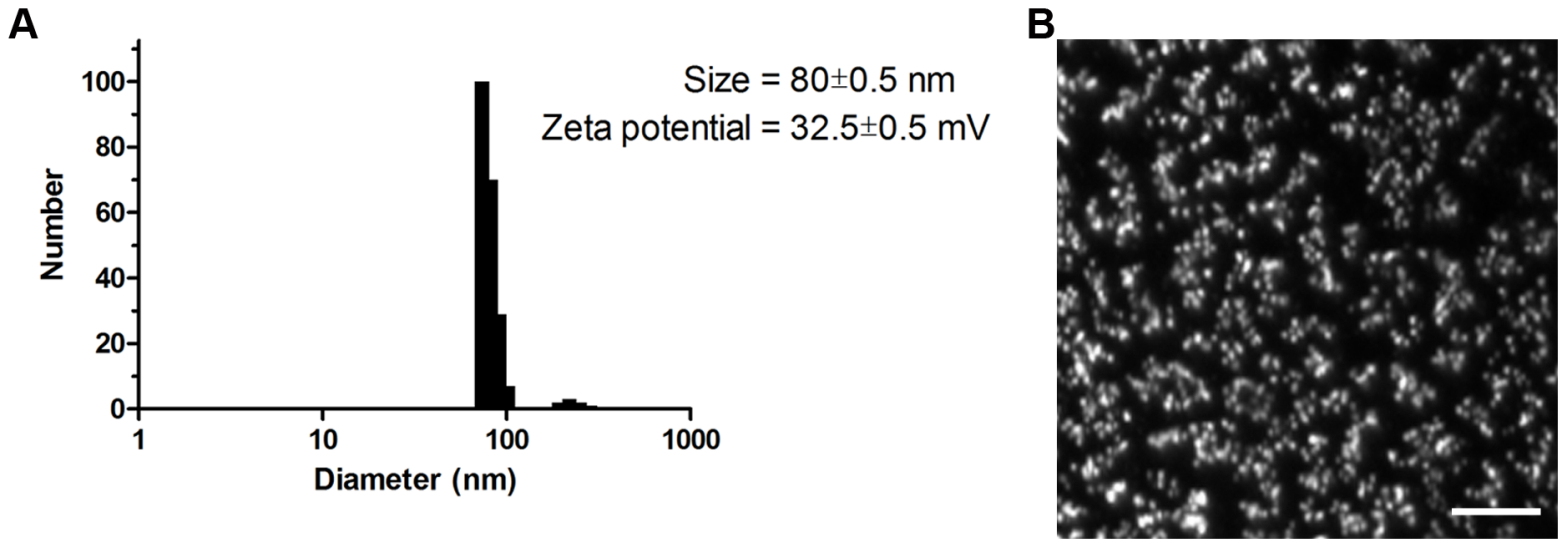
Particle characterization of the 599/siCIP2A complex. (**A**) Size distribution and zeta potential of the 599 peptide complexed with siCIP2A at a 50∶1 peptide-to-siRNA molar ratio 20 minutes after formulation in water. (**B**) Darkfield-based optical microscopy image of the 599 peptide complexed with siCIP2A at a 50∶1 peptide-to-siRNA molar ratio 20 minutes after formulation in water. Scale bar: 10,000 nm.

To assess the 599 peptide in terms of particle size and surface charge after complexation with siCIP2A both dynamic light scattering and zeta potential measurements were performed (**Fig. 4A**). Based on the measurements, 96% of the particles formed were centered at 74 nm and 4% at 220 nm by number-weighted size distribution, with the average particle size of the major population being 80±0.5 nm. The zeta potential of the 599/siCIP2A complex was determined to be positive with a value of 32.5±0.5 mV. The 599/siCIP2A complex was also visualized using darkfield-based optical illumination (**Fig. 4B**). Using this imaging technology the 599/siCIP2A complex was found to exhibit fairly uniform spherical structures.

**Figure 5:**
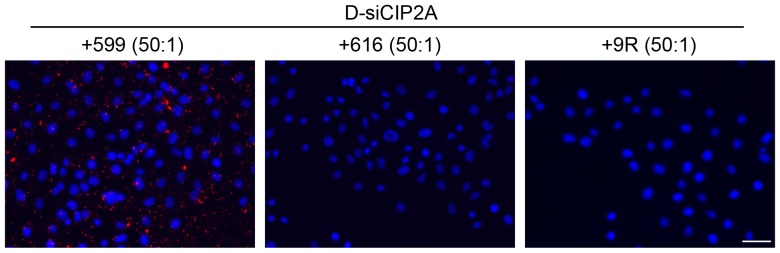
Both the endosome-disruptive and cationic cell-penetrating nona(D-arginine) residue portions of the 599 peptide are required for siRNA delivery into oral cancer cells. Fluorescence microscopy analyses of CAL 27 oral cancer cells incubated for 2 hours with DY547-conjugated siRNA targeting CIP2A (D-siCIP2A; red) complexed to peptides 599, 616, and 9R at a 50∶1 peptide-to-siRNA molar ratio. Nuclei (blue) were counterstained with DAPI. Scale bar: 50 µm.

To test the importance of the two core components (i.e. the endosome-disruptive and cationic cell-penetrating nona(D-arginine) residue portions) in conferring the ability of the 599 peptide to deliver siRNAs into cells, two additional peptides were synthesized consisting of either the endosome-disruptive sequence (616) or the cationic cell-penetrating nona(D-arginine) residues alone (9R). Upon treatment of CAL 27 cells with either the 599, 616, or 9R peptides in complex with D-siCIP2A at a 50∶1 P:N ratio, fluorescence microscopy analyses revealed that only the intact 599 peptide with both the endosome-disruptive and cationic cell-penetrating nona(D-arginine) residue portions could mediate delivery of siRNAs into cells, whereas both the 616 and 9R peptides had no apparent effect on siRNA delivery 2 hours post-treatment (**Fig. 5**).

The failure of the 616 peptide to deliver siRNAs into cells was most likely due to its inability to bind siRNAs even at P:N ratios as high as 100∶1 (data not shown). However, the result with the 9R peptide was somewhat surprising, since it was demonstrated to effectively bind siRNAs at a 50∶1 P:N ratio based on an agarose gel shift assay (**Fig. S1A**) and could form particles with an average size of 58.2±0.2 nm and a zeta potential value of 25.8±0.5 mV (**Fig. S1B**). Because paraformaldehyde fixation of cells can result in the artifactual redistribution of (Arg)_9_ peptides [27], it was possible that the cellular distribution of the 9R/D-siCIP2A complex may have been affected by this chemical fixative. Therefore, the uptake of D-siCIP2A was also assessed using the fixation-independent quantitative approach which similarly found that the 9R peptide, ranging from 0.1 to 100-fold molar excess of siRNAs, was incapable of mediating siRNA delivery into cells and was indistinguishable from cells treated with D-siCIP2A alone (**Fig. S1C**).

### The 599 Peptide Mediates CIP2A Gene Silencing in Oral Cancer Cells

**Figure 6:**
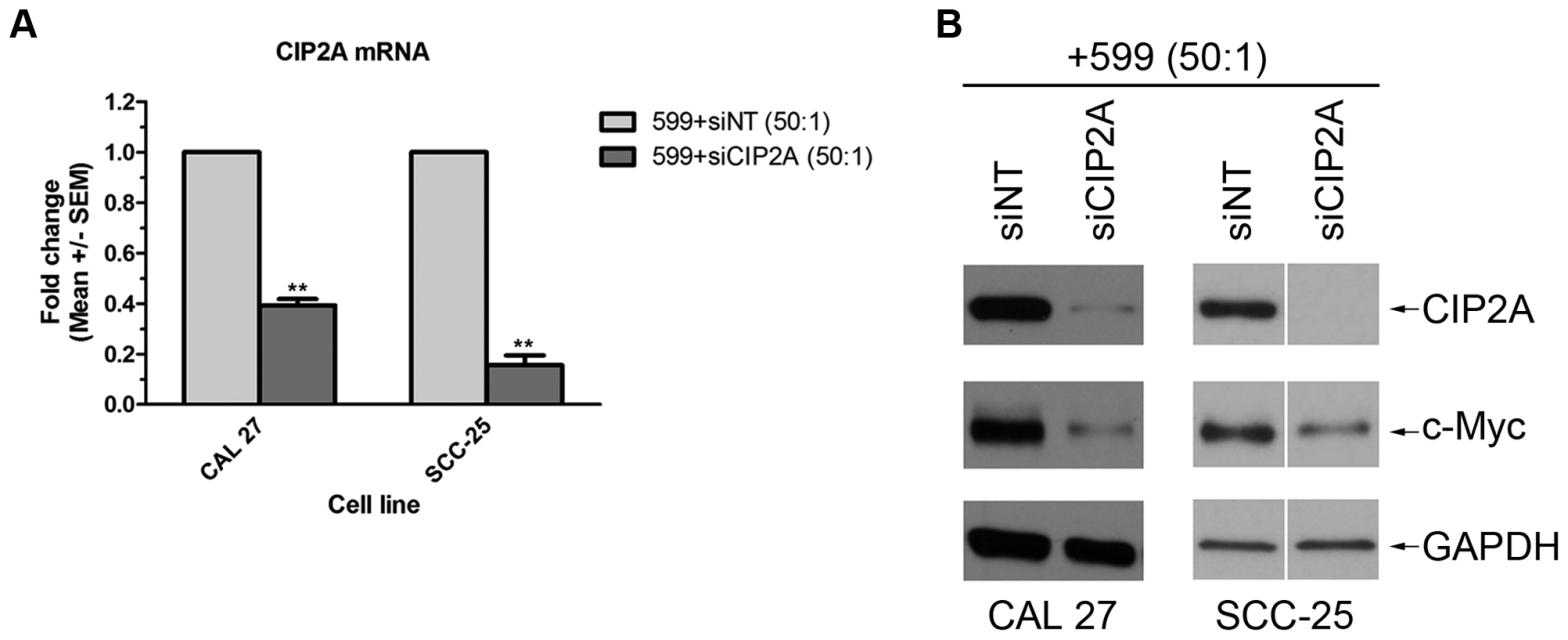
The 599 peptide mediates CIP2A gene silencing and subsequent destabilization of the c-Myc oncoprotein in oral cancer cells. (**A**) Real-time PCR analysis of CIP2A mRNA levels in CAL 27 and SCC-25 oral cancer cells 48 hours post-treatment with 599 peptide complexed at a 50∶1 peptide-to-siRNA molar ratio to 100 nM of siRNA targeting CIP2A (siCIP2A) compared with control non-targeting siRNA (siNT). The CIP2A mRNA levels were normalized to 18S rRNA. Data are mean ± SEM of three separate experiments performed in triplicate, where **P<0.01 compared to siNT treated cells (Student’s t test). (**B**) Western blot analyses of CIP2A and c-Myc protein expression levels in CAL 27 and SCC-25 oral cancer cells 48 hours post-treatment with 599 peptide complexed to either 100 nM of siNT or siCIP2A at a 50∶1 peptide-to-siRNA molar ratio. GAPDH protein levels were monitored to ensure equal loading of samples.

For the 599 peptide to be considered an effective delivery vehicle for siRNA-based therapeutics, it must be capable of delivering functional siRNAs into cells that can silence their intended gene target. To assess the functionality of the 599/siRNA complex, two oral cancer cell lines, CAL 27 and SCC-25, were treated with the 599 peptide complexed to either siCIP2A or a siNT control at a 50∶1 P:N ratio, after which both the CIP2A mRNA and protein levels were assessed 48 hours post-treatment by real-time PCR and Western blot analyses, respectively. Quantitation by real-time PCR demonstrated a significant knockdown in CIP2A mRNA levels in both oral cancer cell lines treated with the 599/siCIP2A complex compared to control 599/siNT treated cells (**Fig. 6A**). More specifically, a ∼60% and ∼85% reduction in CIP2A mRNA levels was observed in CAL 27 and SCC-25 oral cancer cell lines, respectively. Furthermore, Western blot analyses confirmed the ability of the 599 peptide to mediate delivery of functional siRNAs in both cell lines by demonstrating the suppression of CIP2A protein levels 48 hours post-treatment with the 599/siCIP2A complex compared to control 599/siNT treated cells (**Fig. 6B**). Of note, the protein levels of the oncogenic transcription factor c-Myc were also assessed because CIP2A is known to regulate the stability of this protein [25]. Western blot analyses confirmed that silencing of CIP2A caused destabilization of c-Myc in both cell lines (**Fig. 6B**).

### Characterization of the 599/siCIP2A Complex-Mediated RNAi Response

**Figure 7:**
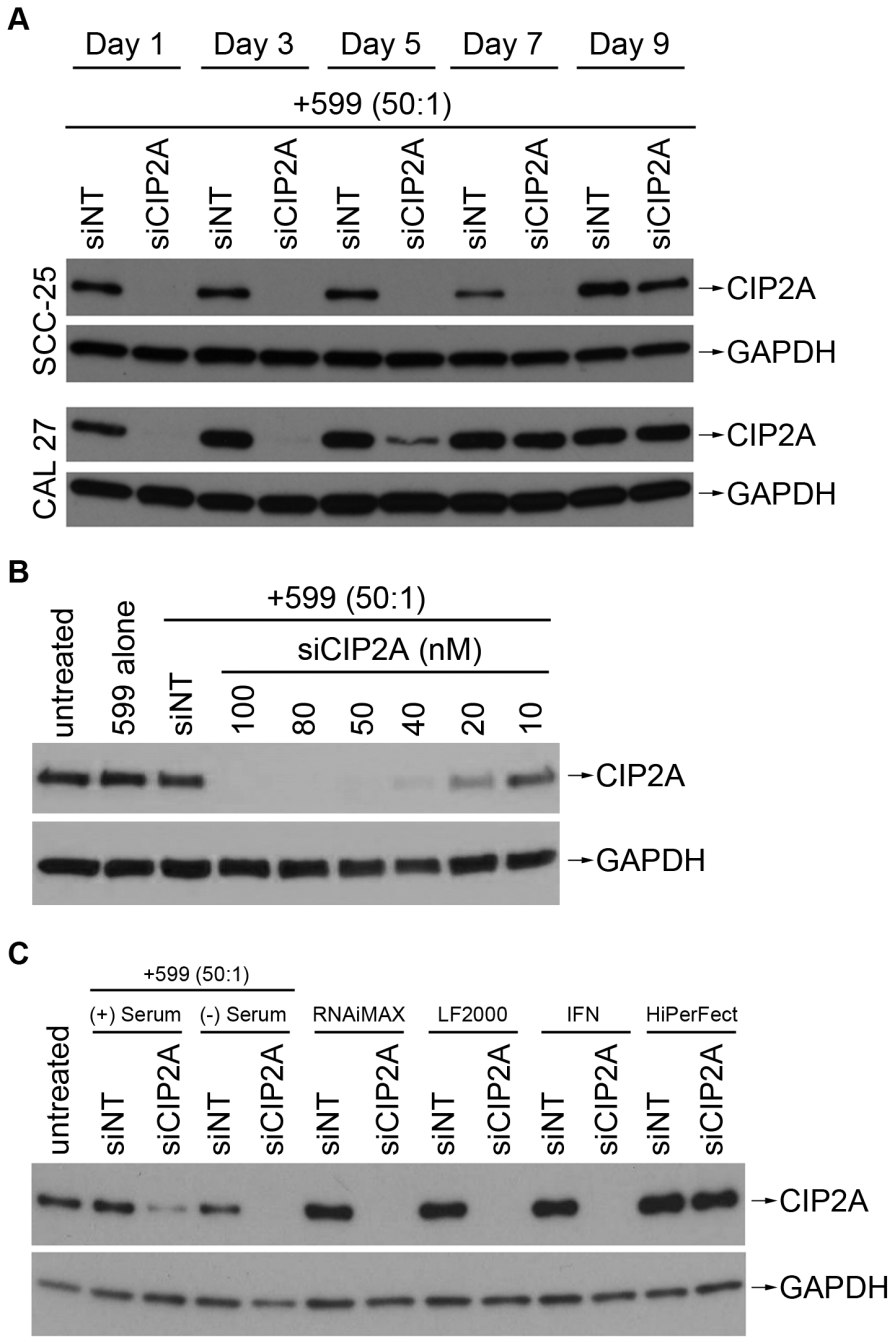
Characterization of 599 peptide-mediated RNAi responses in oral cancer cells. (**A**) Western blot analyses of CIP2A protein expression levels in CAL 27 and SCC-25 oral cancer cells 1, 3, 5, 7, and 9 days post-treatment with 599 peptide complexed to either 100 nM of control non-targeting siRNA (siNT) or siRNA targeting CIP2A (siCIP2A) at a 50∶1 peptide-to-siRNA molar ratio. GAPDH protein levels were monitored to ensure equal loading of samples. (**B**) Western blot analysis of CIP2A protein knockdown in SCC-25 oral cancer cells 48 hours post-treatment with 599 peptide alone (5 µM) or complexed at a 50∶1 peptide-to-siRNA molar ratio to either siNT (100 nM) or decreasing concentrations of siCIP2A. The CIP2A protein levels in untreated cells were also analyzed for comparison. GAPDH protein levels were monitored to ensure equal loading of samples. (**C**) Western blot analyses of CIP2A protein knockdown in SCC-25 oral cancer cells 48 hours post-treatment with 599 peptide complexed to either 100 nM of siNT or siCIP2A at a 50∶1 peptide-to-siRNA molar ratio, in the presence (+) or absence (-) of serum, compared to untreated cells or cells treated with commercial transfection reagents Lipofectamine® RNAiMAX (RNAiMAX), Lipofectamine® 2000 (LF2000), INTERFERin™ (IFN), and HiPerFect®. GAPDH protein levels were monitored to ensure equal loading of samples.

In an effort to further characterize 599 peptide-mediated silencing of CIP2A in oral cancer cells the duration of gene silencing, dose-dependent gene silencing, and activity of silencing in serum were also assessed. The successful implementation of RNAi as a form of therapy depends on these three key features. Thus, in an effort to determine the persistence of 599 peptide-mediated silencing of CIP2A in oral cancer cells over time, a Western blot analyses of CIP2A protein expression levels in CAL 27 and SCC-25 oral cancer cells 1, 3, 5, 7, and 9 days post-treatment with 599 peptide complexed to either siNT or siCIP2A at a 50∶1 peptide-to-siRNA molar ratio were performed (**Fig. 7A**). After a single treatment with 599/siCIP2A, the silencing effect was observed to last for 5 days in CAL-27 cells and up to 9 days in SCC-25 cells with maximum silencing occurring for 3 and 7 days, respectively. In dose-response experiments in SCC-25 cells (**Fig. 7B**), the 599 peptide was observed to confer siCIP2A mediated silencing dose-dependently with siRNA concentrations ≥50 nM conferring ∼100% CIP2A protein knockdown and siRNA concentrations as low as 20 nM still having measurable silencing effects. Similar results were observed in CAL 27 cells (data not shown), except that higher siRNA concentrations (≥80 nM) were needed to confer at best ∼90% CIP2A protein silencing. Lastly, because the stability of siRNAs is an important concern in RNAi-therapy, 599 peptide-mediated CIP2A silencing was assessed in the absence and presence of serum and compared to several commercially available standard cationic lipid-based transfection reagents (**Fig. 7C**). Upon treating SCC-25 cells with 599/siCIP2A, ∼95% CIP2A protein was knocked down in the absence of an initial input of serum and was comparable to CIP2A silencing observed for all cationic lipid-based transfection reagents tested (>99%) except HiPerFect® which exhibited only a ∼30% reduction in protein levels. Of significance, however, is that even in the presence of serum, the 599/CIP2A complex could still produce a very effective RNAi response at ∼70% CIP2A protein knockdown. The quantitation of these data were based on the densitometric analyses of three independent Western blot experiments using Image J software [28].

### The 599 Peptide-Mediated Silencing of CIP2A Decreases Oral Cancer Cell Invasiveness and Anchorage-Independent Growth

**Figure 8:**
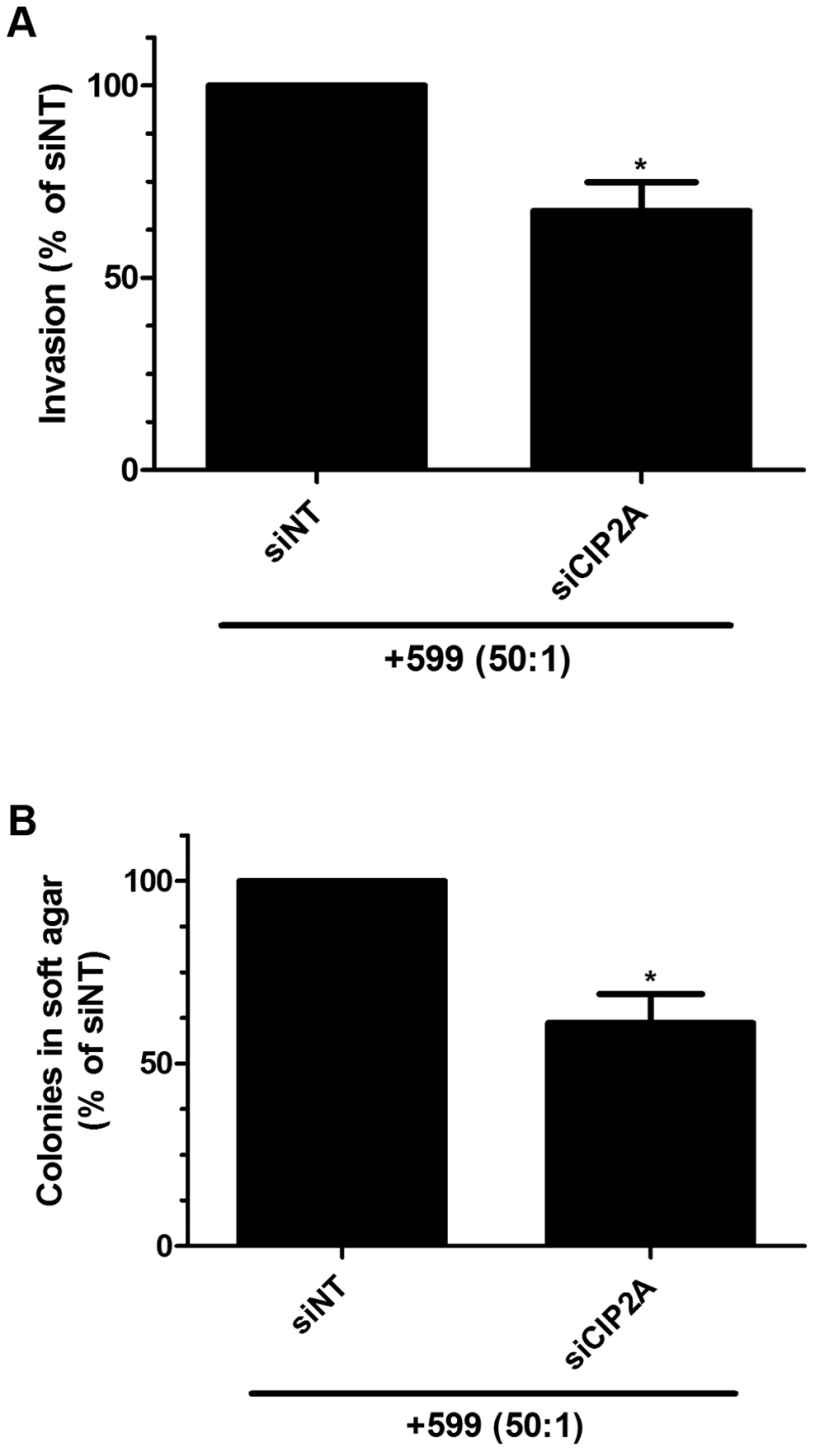
The 599 peptide-mediated silencing of CIP2A decreases oral cancer cell invasiveness and anchorage-independent growth. (**A**) Quantitation of the percentage of invading SCC-25 oral cancer cells after treatment with 599 peptide complexed at a 50∶1 peptide-to-siRNA molar ratio to 100 nM of siRNA targeting CIP2A (siCIP2A) compared with control non-targeting siRNA (siNT). Data are mean ± SEM of four separate experiments, where *P<0.05 compared to siNT treated cells (Student’s t test). (**B**) Anchorage-independent growth of SCC-25 oral cancer cells after treatment with 599 peptide complexed at a 50∶1 peptide-to-siRNA molar ratio to 100 nM of siCIP2A compared with control siNT. Data are mean ± SEM of four separate experiments, where *P<0.05 compared to siNT treated cells (Student’s t test).

Previously published literature has demonstrated that CIP2A silencing in cancer cells derived from different tissues, such as HNSCCs, renal cell carcinomas, and breast cancer can have effects on cancer cell invasion and anchorage-independent growth [25], [29], [30]. Thus, to further demonstrate the potential usefulness of the 599 peptide in siRNA-based therapeutics for oral cancer, we tested whether 599 peptide-mediated silencing of CIP2A could affect the invasiveness and anchorage-independent growth properties of oral cancer cells. Due to the fact that CAL 27 cells exhibited only a brief silencing effect and do not grow well in semi-solid medium [31], the 599 peptide-mediated CIP2A silencing effects on cell invasion and anchorage-independent growth were only tested in SCC-25 cells. Upon treatment of SCC-25 cells with the 599 peptide complexed to siCIP2A, we observed a significant ∼33% inhibition in cell invasiveness, compared to control 599/siNT treated cells (**Fig. 8A**). Moreover, 599 peptide-mediated silencing of CIP2A in SCC-25 cells significantly reduced anchorage-independent growth by ∼39% compared to control 599/siNT treated cells (**Fig. 8B**). Together, these results demonstrated the ability of the 599 peptide to function as an effective delivery vehicle for siRNA-based oral cancer therapeutics.

## Discussion

For RNAi-based technologies to be effective therapies, they require the efficient intracellular delivery of therapeutic siRNAs into the cytoplasm of cells. However, because most delivery approaches traffic siRNA cargo into cells via endocytosis, the entrapment of these molecules within the endocytic vesicles has become a major limiting step [11], [12] and thus a barrier towards the effective use of RNAi-based therapies. To circumvent this problem, we designed and tested a peptide, termed 599, that consisted in part the sequences of both a highly efficient CPP and the endosome-destabilizing INF-7 peptide that when combined would enable effective delivery of complexed siRNA cargo into cells. Results from our study demonstrated that the 599 peptide could increase the intracellular bioavailability of siRNAs in oral cancer cells and effectively mediate the silencing of the targeted CIP2A oncoprotein with consequent inhibition of oral cancer cell invasiveness and anchorage-independent growth.

Because covalent conjugation of siRNAs to CPPs can result in the inefficient delivery of functional siRNAs to cells [18], [32], [33], the 599 peptide was designed to include cationic nona(D-arginine) residues that would function not only as the CPP component, but also enable non-covalent binding of negatively charged siRNA molecules to the peptide via charged interactions. Agarose gel shift assays confirmed that the 599 peptide could complex to siRNA molecules and that the binding between the 599 peptide and siRNAs appeared to be dependent on the nona(D-arginine) residues. This was particularly evident based on the fact that the 616 peptide, which comprised only the highly anionic INF-7 peptide sequence, could not bind siRNAs.

Investigation into whether the 599 peptide-siRNA complex formation was sufficient to induce the internalization of the siRNAs into cells, revealed that increasing the P:N ratios would result in increased siRNA uptake into cells after two hours treatment compared to cells treated with INTERFERin™ or naked siRNA. These findings were consistent with several other studies that similarly found that increasing the concentrations of specific CPPs increased their siRNA uptake ability [20], [23]. The increased uptake of siRNAs into cells most likely occurred at higher P:N ratios based on several factors. First, because the higher P:N ratios were more effective at binding free siRNAs in solution, as was evident through the agarose gel shift assays, this in-part would have contributed to larger quantities of siRNAs being internalized into cells. Second, because treatment of cells with high extracellular concentrations of CPPs has been reported to induce their internalization into cells via activation of endocytosis [34], the increased uptake of siRNAs into cells at higher P:N ratios may have also been attributed to the presence of higher amounts of the 599 peptide which would more effectively trigger endocytosis of the complex.

CPP-mediated internalization into cells is driven by cationic residues found within their sequence and this process has been reported to occur through various forms of endocytosis [18]. In our study, the 599 peptide was observed to mediate the internalization of siRNAs within the first two hours to endocytic vesicles and removal of the nona(D-arginines) from the 599 peptide, as represented by the 616 peptide, confirmed the requirement of cationic residues for the delivery of siRNA cargo. Surprisingly, the 9R peptide which consisted only of cationic nona(D-arginine) residues was similarly incapable of delivering siRNAs into cells, even though it could form complexes with siRNAs. One possible explanation for this contradictory effect could be based on a recent study that found that cells exhibit a preferential uptake of L- versus D-amino acid CPPs with chirality being an important determinant in the capacity of CPP endocytic uptake [35]. In fact, the study demonstrated that nona(D-arginine) peptides were less efficiently internalized into cells compared to their L-counterparts and appeared to accumulate more at the plasma membrane rather than intracellularly [35]. Thus, the 9R peptide in our study most likely could not deliver complexed siRNAs into cells because it was itself incapable of cellular uptake based on its D-amino acid stereochemistry and thus, could explain why in a study by Kim *et al.* [22] complexing siRNAs with nona(D-arginine) peptides alone could not induce silencing of their targeted gene. Interestingly, in this same study, Kim *et al.* also reported that attaching the hydrophobic lipid anchor cholesterol to nona(D-arginine) residues could both enhance the cellular uptake of plasmid DNA and induce silencing of their target gene [22]. In fact, several reports have theorized that combining hydrophobic moieties with cationic agents can produce synergistic effects that enhance the uptake of nucleic acid cargo by either increasing the adsorption of the nucleic acid/carrier complex to the cell surface thereby promoting a greater degree of internalization via endocytosis or causing the compaction of the nucleic acid/carrier complexes [22], [36], [37], [38], [39], [40]. Although, the precise mechanism of how the 599 peptide mediates enhanced delivery of siRNAs still remains to be determined, it is interesting to note that the INF-7 peptide sequence built within the 599 peptide comprises 50% hydrophobic amino acid residues. Thus, one could speculate that the reason the 599 peptide has the ability to deliver siRNAs into cells is because the INF-7 peptide sequence not only has the ability to disrupt endosomal membranes [15], but it also comprises a hydrophobic component that when combined with cationic nona(D-arginines) could confer a synergistic effect that allows the 599 peptide the ability to more effectively deliver siRNA cargo into cells. This capacity to enhance the intracellular bioavailability of siRNAs by the 599 peptide was made evident by its ability to induce significant silencing of the targeted CIP2A oncogene with the consequent destabilization of the oncogenic transcription factor c-Myc 48 hours post-treatment.

The finding that the 599 peptide could mediate the delivery of functional siRNAs into cells was particularly significant because it revealed its potential applicability as a delivery vehicle for siRNA-based therapeutics. In fact, the measured significant inhibition in both cell invasiveness and anchorage-independent growth of SCC-25 cells upon treatment with the 599 peptide-siCIP2A complex compared to control treated cells demonstrated its usefulness for siRNA-based therapeutics, in particular for oral cancer. Although these studies were performed *in vitro*, the 599/siRNA complex does have the potential to also succeed in *in vivo* applications based on several factors. First, it is a simple formulation (a stable complex can be made within minutes in water by simply mixing the 599 peptide with siRNAs); therefore, it would not require time-consuming and expensive particle formulation procedures as observed in other effective *in vivo* siRNA delivery strategies that necessitate more complicated particle formulations [41]. Second, with the hydrodynamic mean diameter of the 599/siCIP2A complex being on average 80 nm, this size is well below particle size cutoffs required for efficient intracellular internalization via various endocytic routes [42], comparable to sizes of other efficient siRNA nanoparticle formulations (<200 nm) [43], and within the particle size range (20–200 nm) necessary for effective retention in tumor tissue [44]. Third, the 599/siCIP2A complex has no associated long-term cytotoxicities and its silencing activity is well preserved in serum. The fact that the 599 peptide could mediate a ∼70% CIP2A knockdown even in the presence of serum with its simple peptide design, begs one to question what improvements could be made with only a few additional simple chemical modifications. For instance, it would be interesting to examine whether changing the chirality of the D-arginines within the 599 peptide to L-arginines would enhance the uptake of the 599/siRNA complex resulting in a stronger RNAi response in the presence of serum. Moreover, to further improve upon this design, one could also change the chirality of the INF-7 peptide sequence while retaining the nona(L-arginines) to make the peptide more protease resistant and thus more stable in the presence of serum. Additionally, because the INF-7 peptide is an amphipathic peptide [14], it would also be worthwhile to test whether adding a stearyl moiety to its N-terminus would improve its activity in serum. Recently, the addition of a stearic acid moiety to an amphipathic CPP was demonstrated to improve the activity of the CPP in serum and make more efficient the delivery of siRNAs in cell culture and systemically *in vivo* [43], [45]. Of note, chemically modified siRNAs were not used in our studies; therefore, there is also the possibility that one could significantly improve the RNAi response even at lower siRNA doses by using optimized formulations of the 599 peptide complexed to chemically stabilized siRNAs. Lastly, because OSCCs are readily accessible for treatment within the oral cavity, one strategy to retain the very effective silencing observed for the 599/siCIP2A complex in the absence of serum that was in parity with the effects observed for well-established commercially available cationic lipid-based transfection reagents, would be to administer the complex via a direct intratumoral injection. The advantage of this form of administration is that this route of dosing does not require as much protection of siRNAs from nuclease degradation compared to systemic administrations [8], thus potentially limiting the loss of RNAi activity mediated by the 599/siCIP2A complex in an *in vivo* setting.

In conclusion, our data collectively demonstrate that the 599 peptide, designed to comprise both synthetic influenza virus-derived fusogenic peptide sequences and cationic CPP nona(D-arginine) residues, can be used to enhance the intracellular delivery and bioavailability of siRNAs via its direct complexation with these molecules. Moreover, the ability of the 599 peptide to mediate silencing of the CIP2A oncogene resulting in decreased oral cancer cell invasiveness and anchorage-independent growth demonstrate the therapeutic relevance of this delivery vehicle. However, future *in vivo* studies using mouse xenograft floor-of-mouth tumors will be needed to more accurately assess the therapeutic potential of the 599 peptide in mediating siRNA-based therapeutics for oral cancer and its prospective applicability in clinical settings.

## Materials and Methods

### Peptide Synthesis

Peptides 599 (GLFEAIEGFIENGWEGMIDGWYGGGGRRRRRRRRRK), 616 (GLFEAIEGFIENGWEGMIDGWYGK), and 9R (RRRRRRRRR) were synthesized and purified (>95% purity) by high-performance liquid chromatography at GenScript USA Inc. (Piscataway, NJ). The 599 and 616 peptides were also biotinylated on the lysine residue and 9R was biotinylated at the N-terminus. In peptides 599 and 9R, the nine arginine residues were D-arginine.

### siRNAs

The siRNA targeting CIP2A (siCIP2A) was previously described (CIP2A.1 siRNA) [25] and synthesized by Thermo Fisher Scientific Dharmacon (Lafayette, CO). Additionally, a siCIP2A with a DY547 label at the 3′-end of the anti-sense strand (D-siCIP2A) was also synthesized and used for some experiments. The control siGENOME Non-Targeting siRNA #2 (siNT2) and #5 (siNT5) were purchased from Thermo Fisher Scientific Dharmacon.

### Cell Culture

Human tongue squamous cell carcinoma (SCC) cell lines CAL 27 and SCC-25 were purchased from American Type Culture Collection (ATCC, Manassas, VA). Each of the cell lines was cultured in ATCC-specified complete growth media in a 37°C incubator with 5% CO_2_.

### Agarose Gel Shift Assay

30 pmol of siCIP2A was incubated with the 599 or 9R peptide at 50∶1, 20∶1, 10∶1, and 1∶1 molar ratios of peptide-to-siRNA at room temperature (RT) for 15 minutes. Afterwards, the samples were subjected to electrophoresis on a 4% agarose gel and stained with ethidium bromide. siCIP2A without added peptide served as a control. The Ambion® pUC19 DNA - Sau3A I digest DNA ladder was purchased from Life Technologies (Grand Island, NY).

### Peptide-Mediated siRNA Delivery and CIP2A Silencing in Oral Cancer Cell Lines

100 nM of D-siCIP2A was incubated either alone or with the 599 peptide at 50∶1, 20∶1, and 1∶1 molar ratios of peptide-to-siRNA for 15 minutes at RT in Opti-MEM® I Reduced Serum Media (Life Technologies). Concurrently, CAL 27 cells grown to a confluency of ∼60% on Collagen Type I coated 8-chamber slides (BD Biosciences, San Jose, CA) were rinsed three times with Opti-MEM® media. Afterwards, the cells were incubated with either siRNA alone or peptide/siRNA solutions for 2 hours before being processed and examined by fluorescence microscopy. For experiments involving siRNA delivery comparisons between the 599, 616, and 9R peptides, 100 nM of D-siCIP2A was used at a 50∶1 molar ratio of peptide-to-siRNA in Opti-MEM® media. Lastly, for CIP2A mRNA and protein silencing experiments, both 96 and 12-well plate formats were used, respectively. In these experiments, 125 nM of either siNT2 or siCIP2A were initially incubated with the 599 peptide at a 50∶1 molar ratio of peptide-to-siRNA for 15 minutes at RT in Opti-MEM® media. Afterwards, the peptide/siRNA solutions were added to either CAL 27 or SCC-25 cells grown to a confluency of ∼60% and incubated for 4 hours, prior to adjusting the siRNA and fetal bovine serum (FBS) concentrations to 100 nM and 10%, respectively, by adding 50% FBS, Opti-MEM® media. For the dose-response experiments, peptide/siRNA solutions consisting of 12.5–125 nM of siCIP2A complexed at a 50∶1 peptide-to-siRNA molar ratio in Opti-MEM® media were initially added to the cells and incubated for 4 hours, prior to adjusting the siRNA and FBS concentrations to 10–100 nM and 10%, respectively. For serum experiments, immediately after complex formation in serum-free media, 50% FBS, Opti-MEM® media was added to adjust the siRNA and FBS concentrations to 100 nM and 10%, respectively, after which the peptide/siRNA solutions were then added to cells. 48 hours post-treatment, the total RNA or protein was harvested for subsequent real-time PCR and Western blot analyses. Protein lysates were also collected 1, 3, 5, 7, and 9 days post-treatment to determine the duration of 599 peptide-mediated silencing by Western blot analysis. As controls for determining the efficacy of 599 peptide-mediated CIP2A silencing, cells were also transfected with 100 nM of siRNA using Lipofectamine® RNAiMAX (Life Technologies), Lipofectamine® 2000 (Life Technologies), HiPerFect® (Qiagen, Valencia, CA), and INTERFERin® (Polyplus-transfection, New York, NY), according to the manufacturer’s instructions. Of note, siNT5 was used as the control non-targeting siRNA for the experiments involving serum stability and the commercially available transfection reagents described above.

### Fluorescence Microscopy

Treated cells seeded on Collagen Type I coated 8-chamber slides (BD Biosciences, San Jose, CA) were rinsed with 1×phosphate-buffered saline (PBS), fixed in 3% paraformaldehyde at RT for 10 minutes and then either mounted immediately with a glass coverslip using VECTASHIELD Mounting Medium with 4′,6-diamidino-2-phenylindole (DAPI, VECTOR Laboratories, Burlingame, CA) or permeabilized with 0.5% Triton X-100 at RT for 5 minutes. For cells that were permeabilized, the slides were subsequently incubated with a rabbit monoclonal anti-EEA1 antibody (1∶200, clone C45B10, Cell Signaling, Danvers, MA) at RT for 1 hour, washed with 1×PBS, and then incubated with the corresponding secondary antibody, Alexa Fluor® 488-conjugated goat anti-rabbit IgG (1∶400, Life Technologies), at RT for 1 hour. Afterwards, coverslips were mounted onto the slides as described above. For live cell imaging, treated cells were washed three times with 1×PBS before being imaged in the presence of PBS. Fluorescence and phase contrast images were obtained using a Zeiss (Thornwood, NY) Axio Observer.D1 microscope. Images of fixed or live cells were taken using a LD A-Plan ×20/0.3 Ph1 objective.

### Quantitative Uptake of Fluorescently Labeled siRNAs

This protocol was adapted from a previously described protocol [20]. Briefly, 60 pmol of D-siCIP2A was incubated either alone or with the 599 or 9R peptides at different peptide-to-siRNA molar ratios for 15 minutes in a total volume of 600 µl of Opti-MEM® media (Life Technologies). CAL 27 cells grown to a confluency of ∼60% on a 24-well plate were then rinsed three times with Opti-MEM® media, after which the cells were incubated with either 500 µl of siRNA alone or peptide/siRNA solutions for 2.5 hours. As controls for the experiments involving the 599 peptide, the cells were also treated with peptide alone (the amount of peptide used was equivalent to that used at a 100∶1 peptide-to-siRNA molar ratio) or transfected with 100 nM of D-siCIP2A using INTERFERin® (Polyplus-transfection), according to the manufacturer’s instructions. After treatment, the cells were rinsed three times with 1×PBS, trypsinated for 10 minutes at 37°C, and then centrifuged at 4°C for 5 minutes at 1,000 g. After centrifugation, the cells were washed with ice cold 1×PBS, centrifuged again, and then lysed in 200 µl of ice cold 0.1 M NaOH. The cell lysates were centrifuged at 4°C for 5 minutes at 10,000 g to remove cell debris and then stored at −80°C. On the day of the fluorescence measurements, the cell lysates were thawed on ice and 100 µl per sample was transferred to a black 96-well plate to measure fluorescence at 530/590 nm using a BioTek (Winooski, VT) Synergy HT plate reader. Fluorescence measurements were converted to the amount of internalized siRNAs using a standard curve that was generated per experiment using known concentrations of D-siCIP2A ranging from 1.6–100 fmol/µl. Afterwards, the amount of internalized siRNA was normalized to the amount of protein which was quantitated using the Pierce BCA Protein Assay Kit (Thermo Fisher Scientific, Rockford, IL).

### Particle Size and Zeta Potential Determination

The hydrodynamic diameter of the 599/siCIP2A and 9R/CIP2A particles formulated at a 50∶1 peptide-to-siRNA molar ratio in water were measured at room temperature by dynamic light scattering using a 90Plus Nanoparticle Size Analyzer (Brookhaven Instruments, Holtsville, NY). Zeta potential was measured using the same instrument.

### Darkfield-Based Optical Microscopy

After formulating the 599/siCIP2A particles at a 50∶1 peptide-to-siRNA molar ratio in water, 2 µl was pipetted onto a glass slide, and the spot was allowed to air dry. Images of the particles were then collected using an Olympus (Center Valley, PA) upright BX51 microscope outfitted with a CytoViva (Auburn, AL) Hyperspectral Imaging System and oil immersion darkfield condenser. All images were collected using a 40×LUC PlanFL N air objective (NA 0.60).

### Real-Time PCR

For CIP2A mRNA and 18S rRNA quantitation, total RNA was harvested and reverse-transcribed using the TaqMan® Fast Cells-to-CT™ kit (Life Technologies). Afterwards, quantitative real-time PCR was performed in an Applied Biosystems® StepOnePlus™ Real-Time PCR machine (Life Technologies) using predesigned TaqMan® Gene Expression Assays (Life Technologies) for CIP2A (Hs00405413_m1) and 18S (4310893E) combined with TaqMan® Fast Universal PCR Master Mix (Life Technologies), according to the manufacturer’s instructions.

### Western Blot Analysis

Treated cells were washed with 1×PBS and lysed using ice cold RIPA buffer (50 mM Tris-HCl, pH 7.4, 150 mM NaCl, 0.5% sodium deoxycholate, 0.1% SDS, and 1% NP-40) with protease inhibitor cocktail (Thermo Fisher Scientific). The protein lysates were then resolved by SDS-PAGE on a 10% gel and transferred to a PVDF membrane. The PVDF membrane was cut at the 50 and 75-kDa molecular weight protein markers into three pieces (for experiments not involving c-Myc analyses the membrane was only cut at the 75-kDa molecular weight protein marker) and blocked in 5% nonfat dried milk in Tris-HCl-buffered-saline (TBS)-0.1% Tween (pH 7.5) for 1 hour at RT. The top portion of the membrane containing the higher molecular weight proteins was incubated with mouse monoclonal anti-CIP2A antibody (1∶500, clone 2G10-3B5, Santa Cruz Biotechnology, Santa Cruz, CA), the middle portion of the membrane containing proteins ranging from 50 to 75-kDa was incubated with rabbit monoclonal anti-c-Myc antibody (1∶1,000, Cell Signaling), and the bottom portion of the membrane containing the lower molecular weight proteins was incubated with rabbit monoclonal anti-GAPDH antibody (1∶10,000, Cell Signaling) for 1 hour at RT. The membranes were then washed four times with TBS-Tween and incubated with the corresponding secondary antibodies either horseradish peroxidase-conjugated goat anti-mouse (1∶4,000) or anti-rabbit IgG (1∶4,000 or 1∶20,000, SouthernBiotech, Burmingham, AL) for 1 hour at RT. Immunoreactive bands were detected by the SuperSignal Chemiluminescent system (Thermo Fisher Scientific), according to the manufacturer’s instructions.

### Cell Viability Measurements

Long-term cell viability was assessed using the CellTiter 96® AQueous One Solution Cell Proliferation Assay (Promega, Madison, WI). Briefly, 15,000 cells were seeded on 96-well plates several days before treatment. Upon reaching a 50–60% cellular confluency, the cells were either treated in Opti-MEM® media with various concentrations of peptide alone, 125 nM siNT2 alone or in complex with 599 peptide (at different peptide-to-siRNA molar ratios), or transfected with 100 nM siNT2 using INTERFERin® (Polyplus-transfection). Of note, for cells treated with peptide alone, siRNA alone, or in complex with the 599 peptide, the cells were initially incubated for 4 hours, prior to adjusting the siRNA (if added) and FBS concentrations to 100 nM and 10%, respectively, by adding 50% FBS, Opti-MEM® media. After 48 hours treatment/transfection, the cell viability was assayed, according to the manufacturer’s instructions. Absorbance at 490 nm was measured using a BioTek Synergy HT plate reader. Untreated cells were defined as 100% viable.

### Cell Invasion Assay

Cell invasion properties were quantified using the CytoSelect™ 24-Well Cell Invasion Assay kit (Cell Biolabs, San Diego, CA). Briefly, 72 hours post-treatment, SCC-25 cells were trypsinized and serum starved in 0.1% (w/v) BSA, Opti-MEM® media for 24 hours prior to being resuspended in the same media at 2×10^5^ cells/ml. Afterwards, 300 µl of cell suspension was added to the insert pre-coated with a basement membrane matrix layer and 500 µl of 10% FBS, Opti-MEM® media was added to the lower well of the invasion plate. After 48 hours, non-invasive cells were swabbed away from the interior of the inserts and invasive cells adhering to the underside were stained and quantified, according to the manufacturer’s instructions. Absorbance at 560 nm was measured using a BioTek Synergy HT plate reader. Data were normalized to the amount of inputted cells grown on a separate plate for the same period of time.

### Anchorage-Independent Growth Assay

Anchorage independent growth in semisolid media was assessed using the CytoSelect™ 96-Well Cell Transformation Assay (Soft Agar Colony Formation; Cell Biolabs). Briefly, 24 hours post-treatment, SCC-25 cells were resuspended using Versene (Life Technologies) at a concentration of 1.6×10^5^ cells/ml. Following the manufacturer’s protocol, the different semisolid layers were assembled. The top layer of growth media was replenished every 2 days. The assay was carried out for 9 days at which time the agar was solubilized, the cells were lysed, and growth was quantitated with a fluorescent dye. Fluorescence was measured at 485/528 nm using a BioTek Synergy HT plate reader.

## Supporting Information

Figure S1 **Characterization of 9R peptide binding and delivery of siRNAs.** (**A**) An ethidium bromide stained 4% agarose gel shift assay examining the ability of various amounts of the 9R peptide (ranging from 1 to 50-fold molar excess of siRNAs) to form complexes with siCIP2A. siCIP2A, siRNA targeting the CIP2A oncogene; MWM, molecular weight marker (the number of base pairs for each DNA fragment are shown). (**B**) Size distribution and zeta potential of the 9R peptide complexed with siCIP2A at a 50∶1 peptide-to-siRNA molar ratio 20 minutes after formulation in water. (**C**) CAL 27 cells incubated for 2.5 hours with DY547-conjugated siRNA targeting CIP2A (D-siCIP2A) alone or in complex with increasing amounts of 9R peptide (ranging from 0.1 to 100-fold molar excess of siRNAs). As a positive control, the cells were also treated with the 599 peptide complexed to D-siCIP2A at a 50∶1 peptide-to-siRNA molar ratio. The amount of siRNA delivered into cells in pmol per mg of protein is reported with each treatment normalized to D-siCIP2A alone. Data are mean ± SEM of three separate experiments, where ***P<0.001 compared to D-siCIP2A alone treated cells (ANOVA, Dunnett’s Multiple Comparison Test).
(TIF)
