# Supplementary figures and images for: Fusogenic-Oligoarginine Peptide-Mediated Delivery of siRNAs Targeting the CIP2A Oncogene into Oral Cancer Cells
Source: PLoS One. 2013 Sep 3;8(9):e73348. doi: 10.1371/journal.pone.0073348 (PMC3760901; doi:10.1371/journal.pone.0073348)

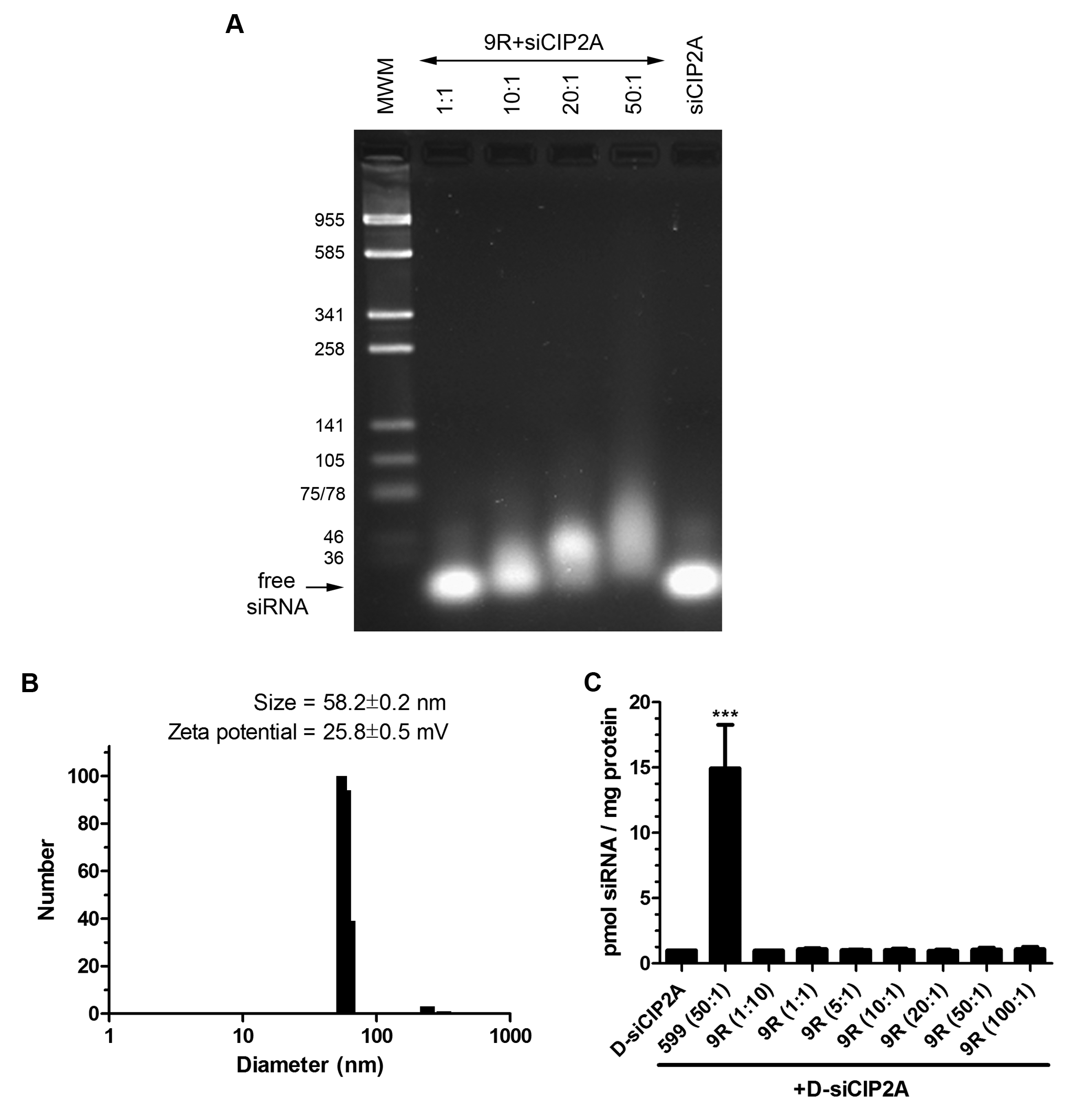

Supplement: Figure S1 — Characterization of 9R peptide binding and delivery of siRNAs. (A) An ethidium bromide stained 4% agarose gel shift assay examining the ability of various amounts of the 9R peptide (ranging from 1 to 50-fold molar excess of siRNAs) to form complexes with siCIP2A. siCIP2A, siRNA targeting the CIP2A oncogene; MWM, molecular weight marker (the number of base pairs for each DNA fragment are shown). (B) Size distribution and zeta potential of the 9R peptide complexed with siCIP2A at a 50∶1 peptide-to-siRNA molar ratio 20 minutes after formulation in water. (C) CAL 27 cells incubated for 2.5 hours with DY547-conjugated siRNA targeting CIP2A (D-siCIP2A) alone or in complex with increasing amounts of 9R peptide (ranging from 0.1 to 100-fold molar excess of siRNAs). As a positive control, the cells were also treated with the 599 peptide complexed to D-siCIP2A at a 50∶1 peptide-to-siRNA molar ratio. The amount of siRNA delivered into cells in pmol per mg of protein is reported with each treatment normalized to D-siCIP2A alone. Data are mean ± SEM of three separate experiments, where ***P<0.001 compared to D-siCIP2A alone treated cells (ANOVA, Dunnett’s Multiple Comparison Test). (TIF) [file pone.0073348.s001.tif]
